# Book Reviews

**Published:** 1892-03

**Authors:** 


### Book Reviews.

---

Annual of the Universal Medical Sciences. A yearly report of the Progress of the General Sanitary Sciences throughout the world. Edited by Chas. E. Sajons, M. D., and seventy associate editors, assisted by over two hundred corresponding editors, collaborators and correspondents. Illustrated with chromo-lithographs, engravings and maps. F. A. Davis, Philadelphia, 1891.

The Annual for '91 is fully up to the standard of its predecessors in every respect and surpasses them in many. The different subjects have been more uniformly well handled than heretofore. The addition of a reference list at the end of each volume is a valuable new feature.

The publishers and engravers have done their work well.

The Annual abundantly deserves the liberal patronage which it has received ; and we bespeak for it the continued success to which its merit as an accurate mirror of current medical literature justlyentitles it.

---

Wounds and Obstruction of the Intestines. Edward Martin, M. D., Instructor in Operative Surgery, University of Pennsylvania, and Hobart A. Hare, M. D., Professor of Therapeutics, Jefferson Medical College, Philadelphia. W. B. Saunders, Philadelphia.

This monograph of one hundred and sixty-four pages was the prize dissertation presented to the Trustees of the Fiske Fund, Rhode Island Medical Society, June, 1890. A partial table of contents includes: Intussusception, volvulus, obstruction from foreign bodies, peritonitis, diagnosis of the various forms of intestinal obstruction, general and special treatment of obstruction, surgical treatment of obstruction, wounds of the intestines, rupture of the intestines, etc. Last of all is a useful, tabulated report of one hundred and thirty cases of gunshot wound of the abdomen. This is the fairest and fullest presentation of these subjects that it has ever been our pleasure to read. It is neither too conservative nor too progressive, and the statements made are both accurate and reliable. The authors have no theories to establish, and hence their combined work is honest and impartial. Any physician will find this work a valuable addition to his library.

---

PULMONARY CONSUMPTION, A NERVOUS DISEASE, FROM A PRACTICAL, CLINICAL AND THERAPEUTIC STANDPOINT. By Thos. J. Mays, M. D., Professor of Diseases of the Chest in the Philadelphia Polyclinic; Visiting Physician to the Rush Hospital for Consumptives, etc. George S. Davis, Detroit, Mich.

The author of this monograph, the title of which must take every one somewhat by surprise, has found the bacillus theory as to the causation of pulmonary phthisis to be unsatisfactory and inadequate. Though generally accepted it does not explain the phenonema of consumption; neither has it been of assistance in our treatment of the disease. With this conviction, Dr. Mays now proposes his neurotic theory which asserts that disturbances of the nervous system play either a "causative or a concomitant role" in the history of phthisis. The author writes well; his arguments are plausible, but in view of the difficult position he attempts to sustain they are far from convincing.

